# Multidisciplinary Approach to the Treatment of Obese Adolescents: Effects on Cardiovascular Risk Factors, Inflammatory Profile, and Neuroendocrine Regulation of Energy Balance

**DOI:** 10.1155/2013/541032

**Published:** 2013-10-27

**Authors:** Ana Raimunda Dâmaso, Aline de Piano, Raquel Munhoz da Silveira Campos, Flávia Campos Corgosinho, Wolfgang Siegfried, Danielle Arisa Caranti, Deborah Cristina Landi Masquio, June Carnier, Priscila de Lima Sanches, Patrícia Leão da Silva, Cláudia Maria Oller Nascimento, Lila Missae Oyama, Alexandre Dâmaso Aguilera Dantas, Marco Túlio de Mello, Sergio Tufik, Lian Tock

**Affiliations:** ^1^Department of Biosciences, Universidade Federal de São Paulo (UNIFESP), Rua Silva Jardim, 136 Térreo, Vila Mathias, 11015-020 Santos, SP, Brazil; ^2^Post-Graduate Program of Nutrition, Escola Paulista de Medicina, Universidade Federal de São Paulo (UNIFESP), Rua Marselhesa 630, Vila Clementino, 04020-060 São Paulo, SP, Brazil; ^3^Obesity Rehabilitation Centre INSULA, Insulaweg, Bischofswiesen, Germany; ^4^Department of Physiology, Universidade Federal de São Paulo (UNIFESP), Rua Botucatu 726, Vila Clementino, 04021-001 São Paulo, SP, Brazil; ^5^Weight Science, Rua Teodoro Sampaio, 744 Cj 98 Jardim America, 05406-000 São Paulo, SP, Brazil; ^6^Psychobiology Department, Universidade Federal de São Paulo (UNIFESP), Rua Botucatu 726, Vila Clementino, 04021-001 São Paulo, SP, Brazil

## Abstract

The prevention of obesity and health concerns related to body fat is a major challenge worldwide. The aim of this study was to investigate the role of a medically supervised, multidisciplinary approach, on reduction in the prevalence of obesity related comorbidities, inflammatory profile, and neuroendocrine regulation of energy balance in a sample of obese adolescents. A total of 97 postpuberty obese adolescents were enrolled in this study. Body composition, neuropeptides, and adipokines were analysed. The metabolic syndrome was defined by the International Diabetes Federation (IDF). The abdominal ultrasonography was performed to measure visceral, subcutaneous fat and hepatic steatosis. All measures were performed at baseline and after one year of therapy. The multidisciplinary management promoted the control of obesity reducing body fat mass. The prevalence of metabolic syndrome, asthma, nonalcoholic fatty liver disease (NAFLD), binge eating, and hyperleptinemia was reduced. An improvement in the inflammatory profile was demonstrated by an increase in anti-inflammatory adiponectin and reduction in proinflammatory adipokines, plasminogen activator inhibitor-1, interleukin-6 concentrations, and in the Lep/Adipo ratio. Moreover, a reduction in the AgRP and an increase in the alfa-MSH were noted. The multidisciplinary approach not only reduced obesity but also is efficacious in cardiovascular risk factors, inflammatory profile, and neuroendocrine regulation of energy balance.

## 1. Introduction

The prevalence of overweight and obesity has increased in many low- and middle-income countries [1]. It is estimated that 50% of the adult Brazilian population is overweight, with an obesity rate of 12.5% and 16.9% for men and women, respectively [2]. Similar trends were observed in adolescents, whereby 21% present some degree of overweight and obesity. Previously, it has been showed that obese adolescents present a high degree of nonalcoholic fatty liver disease (NAFLD), metabolic syndrome, asthma, binge eating symptoms, disruption in the neuroendocrine regulation of energy balance, and other impairments, reducing the effectiveness of weight management [3–9]. 

In obese Brazilian adolescents it was observed that 27.16% present diagnoses of metabolic syndrome (MS). The most frequently altered parameter was HOMA-IR [4, 10]. This result suggests a heightened pro/inflammatory state, considering that insulin resistance (IR) is an independent predictor of alterations in the carotid intima-media thickness (IMTc), a subclinical surround of atherosclerosis in obese adolescents [11]. 

Obesity is a multifactor disease, resulting mainly from sedentary behaviours coupled with inadequate eating habits [12, 13]. Researchers worldwide have been searching for the best strategy to combat obesity [14–16]. Recent data supports the short-term effectiveness and clinical utility of using the interdisciplinary model. Moreover, a multicentre evaluation of an interdisciplinary weight loss program for obesity showed that this kind of intervention is highly effective, not only in obesity but for other related disorders. This data suggests the importance of this type of therapy as a strategy required for sustained success of long-term life treatment [16]. Furthermore, the importance of parenting in the effectiveness of the treatment and the prevention of behaviour problems in the control of obesity and health disorders in children and adolescents was recently demonstrated [17].

The overall result of a multifaceted obesity-management program, addressed by using multidisciplinary teams, in local and international literature is limited. The purpose of this study was to analyse the effects of a one-year weight loss approach on the control of obesity and related comorbidities, including metabolic profile, inflammatory markers, and neuroendocrine regulation of energy balance on a sample of obese adolescents within a medically supervised multidisciplinary obesity-management program. 

## 2. Material and Methods

### 2.1. Conceptual Design of Interdisciplinary Obesity Management

**Figure 1:**
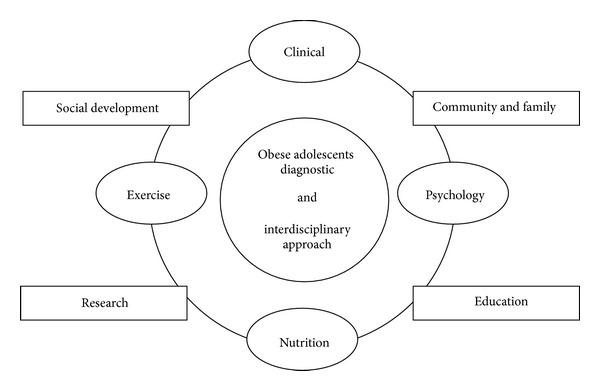
Design conception of interdisciplinary therapy on obesity management in adolescents.

Taking into consideration the multfactorial characteristics of obesity, a multidisciplinary therapy program was developed with the twin aims of combating the main trigger factors of obesity and increasing adherence to, as well as improving responses to, the treatment (Figure 1).

### 2.2. Sample

For this study, a sample of 97 postpuberty obese adolescents (56 girls and 41 boys) was engaged, with ages of 15–19 years, and the sampling method was a consecutive selection of the sample. The study was announced in the media on TV and radio and in journals and the adolescents were selected according to the inclusion and exclusion criteria of the study. The adolescents of this study had the same level of education and mainly belonged to the same social class and ethnicity. These adolescents were recruited from São Paulo, Brazil, and the program ran from January to December during three consecutive years. 

Inclusion criteria were tanner stage five [18], primary obesity, and body mass index (BMI) greater than 30 kg/m² (BMI > 95th percentile of the CDC reference growth charts). Exclusion criteria were age under 14 or over 18 years, the use of birth control pills, cortisone, antiepileptic drugs, history of renal disease, alcohol intake, use of tobacco, secondary obesity due to endocrine disorders, diagnoses of ferritin alteration, autoimmune diseases, and presence of Hepatitis A, B and C virus. 

Only the adolescents who had compliance of 75% in all sections of the therapy were included in the analyses. The main reasons for dropping out (*n* = 20) in the present study were financial and family problems, followed by school and job opportunities. No sex differences were observed in adherence rates. The study was conducted with the principles of the Declaration of Helsinki and was approved by the ethics committee on research at the Universidade Federal de São Paulo (UNIFESP) with the number 0135/04, ClinicalTrials.gov NCT01358773. All procedures were made clear to those responsible for the volunteers and consent for research was agreed. All evaluations were performed at two different times (baseline: beginning and end of therapy: after 1 year of therapy).

### 2.3. Anthropometric Measurements

Weight was measured by the plethysmography scale (BODPOD equipment) where patients wore the minimum clothing possible and height was measured using a stadiometer (Sanny, model ES 2030). After obtaining the data, body mass index (BMI) was calculated by dividing the weight by height squared (kg/m^2^). Body composition, including body fat (percentage and kilograms) and lean mass (percentage and kilograms), was obtained through air displacement plethysmography (BODPOD).

### 2.4. Serum Analysis

Blood samples were collected at the outpatient clinic at approximately 8:00 a.m. after an overnight fast (12 hours). Insulin resistance was assessed by the homeostasis model assessment insulin resistance index (HOMA-IR). HOMA-IR was calculated using the FBG and the immune-reactive insulin (I): (FBG (mg/dL) × I (mU/l))/405. Triglyceride (TG), HDL, low-density lipoprotein (LDL), and very-low-density lipoprotein (VLDL) were analysed using a commercial kit (CELM, Barueri, Brazil). The reference values were HOMA-IR < 2.0 and insulin < 20 *μ*U/mL, as previously described by Schwimmer et al. [19].

The adipokines concentrations were measured using commercial immunoassay kits eBioscience (San Diego, CA, USA) and R&D Systems (Minneapolis, MN, USA) according to manufacturers' manual. The reference values for leptin have been previously described by Gutin et al. [20]. 

### 2.5. Hepatic Steatosis, Visceral and Subcutaneous Adiposity Measurements

The abdominal ultrasonography (US) procedures and the measurements of visceral and subcutaneous fat tissue and fatty liver were performed by the same physician at the baseline time point and following 1 year of therapy. This physician was a specialist in imaging diagnostics. A 3.5-MHz multifrequency transducer (broad band) was used to reduce the risk of misclassification. The intraexamination coefficient of the variation for abdominal US was 0.8%. US measurements of intra-abdominal (visceral) and subcutaneous fat were obtained. US-determined subcutaneous fat was defined as the distance between the skin and external face of the rectus abdominal muscle, and visceral fat was defined as the distance between the internal face of the same muscle and the anterior wall of the aorta. The cut-off points for the definition of visceral obesity by ultrasonography were based on the previous methodological descriptions [21]. In a previous study, it was found that US seemed to be the best alternative method for the assessment of intra-abdominal fat in obese women. US correlated positively with computed tomography (CT). A value of 6.90 cm for visceral fat US determined diagnosed visceral obesity with a specificity of 82.8%, a sensitivity of 69.2%, and a diagnostic concordance of 74% with CT [21].

Steatosis evaluation was performed by abdominal ultrasonography. The definition of ultrasonic fatty liver was based on previously reported diagnostic criteria and detected liver steatosis was classified as grade 1 (liver attenuation slightly less than spleen); grade 2 (more pronounced difference between liver and spleen and intrahepatic vessels not seen or slightly higher attenuation than liver); grade 3 (markedly reduced liver attenuation with a sharp contrast between liver and intrahepatic vessels) [22, 23]. In this study, the group with NAFLD presented some liver steatosis grade diagnosed by abdominal US.

### 2.6. Clinical Intervention

#### 2.6.1. Medical Monitoring

Adolescents underwent a diagnostic clinical evaluation for general health (family history and obesity) and sexual maturation was also assessed. Subsequently, adolescents were examined monthly. Metabolic syndrome was defined using International Diabetes Federation criteria: a waist circumference (WC) greater than 80 cm for girls and 94 cm for boys, high-density lipoprotein (HDL) values ≤50 mg/dL for girls and ≤40 mg/dL for boys, concentrations of triglycerides (TG) higher than 150 mg/dL, blood glucose levels higher than 100 mg/dL, and blood pressures ≥130/85 mmHg [24]. The diagnosis of asthma was made according to ATS guidelines [25]. 

#### 2.6.2. Physical Intervention

During the intervention (1 year), adolescents followed a program of combined training (aerobic/resistance exercise) which included sessions of 60 minutes (30 min aerobic and 30 min resistance) with a frequency of three times a week (180 minutes/week), under the supervision of an exercise physiologist. The aerobic training program was established based on a cardiopulmonary exercise test. The intensity of aerobic training was fixed at the load corresponding to the first ventilatory threshold (50%–70% of oxygen consumption test). During every session of aerobic exercise, heart rate was monitored by a frequency counter. Resistance training was divided into two types, wave (with loads of repeats 15–20, 10–12 and 6–8) and linear (20 replicates), based on exercises that covered large muscle groups of both lower and upper limbs. The program follows the recommendations given by the American College of Sports Medicine [26].

#### 2.6.3. Nutritional Intervention

Food consumption was set at the recommended levels of dietary intake for individuals with low levels of physical activity, based on age, gender, and a balanced diet [27]. No medication or types of supplements were recommended. Once a week, the adolescents had classes on topics related to improved food consumption and they all received individual consultations during the intervention program. At the beginning and after the long-term multidisciplinary therapy, each adolescent filled in a three-day dietary record with the help of his/her parents. These dietary data were transferred to a computer by the same nutritionist, and the nutrient composition was analysed by a PC program developed at the Universidade Federal de São Paulo, Escola Paulista de Medicina (Nutwin software, for Windows, 1.5 version, 2002)—based on Western and local food tables. 

The caloric content was estimated using Dietary Reference Intakes equations, according to low levels of physical activity, based on age and patient gender. The distribution of macronutrients was fat (25–35%), carbohydrate (45–65%), and protein (10–30%). It is important to highlight that food quality was also assessed during nutrition intervention [27, 28].

#### 2.6.4. Psychological Intervention

Diagnoses of psychological problems most commonly associated with obesity, such as depression, body image concerns, anxiety, and lower self-esteem, were assessed through validated questionnaires. During long-term intervention, adolescents were followed up weekly in the one year therapy support group, and if necessary, individual psychological therapy was recommended when behavioural alterations were found (data not shown).

**Figure 2:**
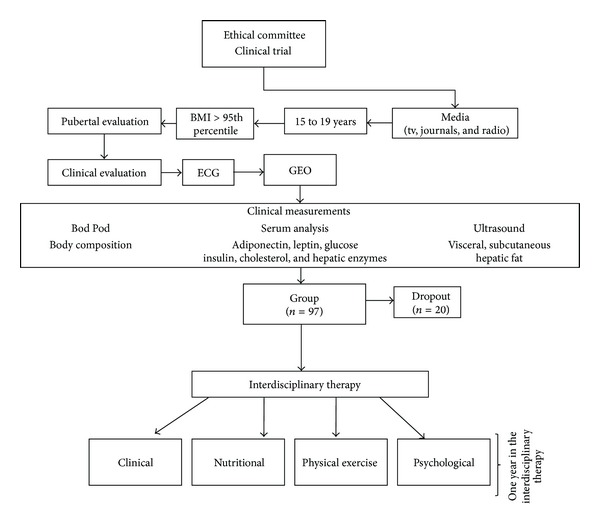
Methodological description.

During psychological therapy, all adolescents completed the Portuguese versions of the BES to verify the symptoms of binge eating and BITE to verify Bulimia symptoms, including the purgative subtype (Figure 2) [29, 30]. 

#### 2.6.5. Statistical Analysis

The data are presented as the mean ± standard deviation (SD), and *P* ≤ 0.05 was considered statistically significant. Distributional assumptions were verified using the Kolmogorov-Smirnov test, and nonparametric methods were performed where appropriate. Adipokines levels were analysed using parametric tests and were expressed as mean ± SD unless otherwise stated. Comparisons between the baseline measurements and the measurements after the weight-loss therapy were made using the paired student's *t*-test or the Wilcoxon signed rank test for nonparametric variables. The prevalence of obesity complications was demonstrated by the frequency table and applied chi-square test (McNemar) analysis. Correlation analysis between the variables was performed using Pearson's correlation test. The delta variation (Δ) was used for the statistical analysis obtained from the difference between the baseline and final values for each variable. All of the analyses were computed using STATISTICA version 6 for Windows. 

## 3. Results

### 3.1. Effects of Therapy on the Prevalence of Related Comorbidities

**Figure 3:**
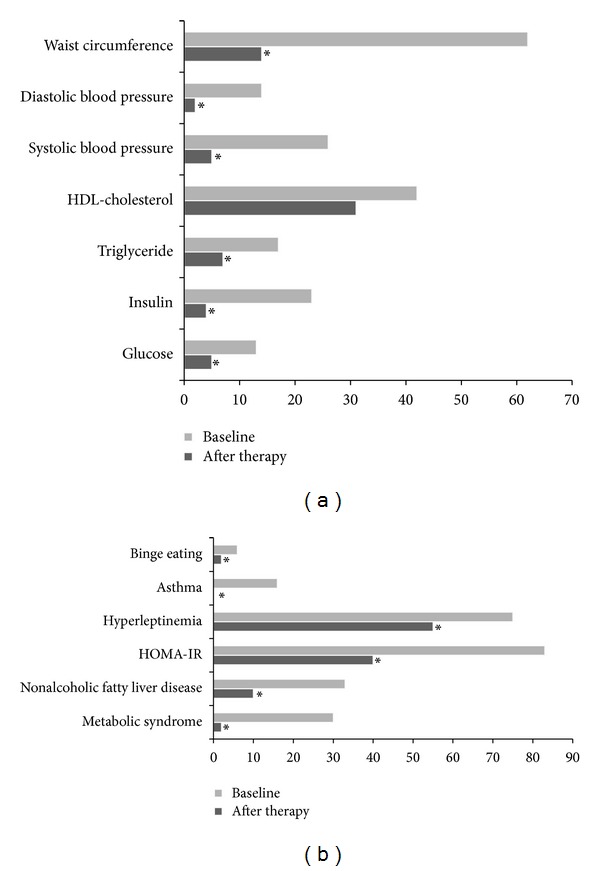
Prevalence of obesity complications: (a) metabolic syndrome factors; (b) comorbidities associated with obesity; *statistical significance, *P* < 0.05.

The interdisciplinary management promoted obesity control, reducing the prevalence of metabolic syndrome, nonalcoholic fatty liver diseases, asthma, insulin resistance, and eating disorders, including symptoms of bulimia nervosa and binge eating disorder (Table 1; Figures 3(a)-3(b)).

### 3.2. Effects of Therapy on Body Composition, Visceral and Subcutaneous Fat and Metabolic Profile

A significant reduction in the body mass, fat mass, visceral fat, subcutaneous fat, waist circumference, and blood pressure was shown. These data were associated with an increased fat free mass, demonstrating an improvement in most risks related to obesity, after one year of interdisciplinary therapy (Table 2). However, it is important to state that only one obese adolescent reached the reference values of body fat mass after therapy, according to the Paediatric Brazilian Association [31]. 

Other important results observed in the present study were the statistical reduction in insulin, triglyceride, LDL, and VLDL cholesterol concentrations. On the other hand, HDL concentration and insulin resistance index (HOMA-IR) were improved (Table 3). However, the glucose concentration did not change the values remaining in the normal concentration. 

### 3.3. Effects of Therapy on the Markers of Neuroendocrine Regulation of Energy Balance

In addition, the weight loss therapy promoted an improvement in the markers of neuroendocrine regulation of energy balance, including a significant reduction in the orexigenic factor AgRP, as well as an increase in the anorexigenic factor *α*-MSH. However, no significant changes in the NPY, MCH, and ghrelin concentrations and NPY/AgRP ratio were observed (Table 4).

### 3.4. Effects of Therapy on the Inflammatory Markers

The inflammatory profile was improved as demonstrated by an increase in the concentration of anti-inflammatory adipokine adiponectin, and a reduction in the proinflammatory adipokines, including plasminogen activator inhibitor-1 and interleukin-6 concentrations. Moreover, the hyperleptinemia was reduced and leptin/adiponectin ratio was improved. However, no changes in TNF-alpha, CRP, and resistin concentrations were observed (Table 4).

### 3.5. Correlation Analysis

Finally, in the correlation analysis of the delta values (Δ) a positive correlation between fat mass with Lep/Adipo; subcutaneous fat with fat mass; plasminogen activator inhibitor-1 with ghrelin was observed. Conversely, negative correlations were observed between fat mass with fat free mass and adiponectin; subcutaneous fat with fat free mass; adiponectin with body mass (Table 5).

## 4. Discussion

The prevention of obesity and health concerns related to excessive body fat are major challenges worldwide, especially considering the effect of childhood and adolescent obesity on our productive population in the near future. Therefore, using a holistic approach and multidisciplinary therapies, addressing related risk factors including a reduction in the prevalence of metabolic syndrome (MS), nonalcoholic fatty liver disease (NAFLD), asthma, and dyslipidemia whilst promoting improved quality of life and health, is valid. 

Corroborating, data using the IDF criteria showed that 70% of obese women had diagnosis to MS. Moreover, an alarming prevalence of MS in childhood was found, suggesting therefore a focus on primary prevention and the promotion of healthy lifestyles. In addition, it was suggested that diet, exercise training, and weight loss provide significant clinical benefits and must be considered as the first line for treating both NAFLD and MS. Together, these results highlight the importance of multidisciplinary approach in early life [32–34]. 

In fact, MS and NAFLD have been implicated in both disruption of neuroendocrine regulation of energy balance and accentuated inflammatory process, which may impair the benefits of weight loss therapy [6, 10, 35]. Therefore, an important finding corroborating this hypothesis is that the balance between orexigenic and anorexigenic factors was improved, since a reduction in AgRP and increase in the alfa-MSH were observed in the present study. This was probably modulated by a significant reduction in the state of hyperleptinemia, a key hormone implicated in the central and peripheral control of energy balance [9]. 

Moreover, a reduction of 27% of body fat, a significant reduction in visceral fat, subcutaneous fat, waist circumference, and an increase in the free fat mass was observed (Table 2). In support of this we showed a negative correlation between delta values of fat mass with free fat mass (*R* = −0.75,  *P* = 0.001) (Table 5). Fujioka et al. showed that the decrease in the visceral/subcutaneous ratio and visceral fat was strongly correlated with the improvement in plasma glucose and lipid metabolism [36]. Furthermore, Lee et al. showed that a reduction in body fat had significant effects on metabolic diseases, including cardiovascular disorders. Thus, this significant reduction in fat mass improves not only life expectancy but also significantly reduces the public sector costs associated with obesity related diseases [37, 38]. In addition, it is important to note that insulin resistance was decreased significantly in the study group (Table 3); however, the comparison between genders showed that the therapy was more effective to reduce insulin and HOMA-IR in males compared with female adolescents. However, the values of insulin and HOMA-IR were higher in male compared with female (data not shown). 

On the other hand, the NPY and MCH were not improved. This may have occurred as a result of the percentage of weight loss (approximately 12% of their body mass), suggesting that these neuropeptides require a massive weight loss to favour changes in concentrations and improve the energy balance. In fact, it was shown previously in another study with obese adolescents that in the beginning of weight loss therapy the NPY concentration was increased as a compensatory adaptation and after a massive weight loss the NPY returns to basal values [39]. The second hypothesis is that in this analysis, the ghrelin concentration was not changed, with the role of this orexigenic hormone in the upregulation of NPY being well established. 

Moreover, the neuroendocrine regulation of energy balance is influenced by PAI-1, since it was demonstrated that this prothrombotic adipokine is involved in the response of NPY concentration in obese adolescents [35]. This was confirmed in the present investigation by a positive correlation between delta values of PAI-1 with ghrelin concentration (*R* = 0.68;  *P* = 0.002) (Table 5). Thus, another important finding in the present investigation was a reduction in PAI-1 and interleukin-6. Supporting this result, the adiponectin was significantly increased, favouring amelioration in the Lep/Adipo ratio, improving the control of subclinical inflammation, commonly associated with obesity. 

The adiponectin concentration is a potent anti-inflammatory adipokine and acts in the regulation of insulin homeostasis, favouring the control of many chronic diseases, including atherosclerosis, hypertension, NAFLD, metabolic syndrome, cardiovascular diseases, thrombosis, and asthma [6, 7, 11, 40]. These data reinforce the importance of the results observed in the present study, mainly the reduction of hyperleptinemia and the increase in the adiponectinemia, promoting the decrease in the Lep/Adipo ratio. In addition, we showed a positive correlation between delta values of Lep/Adipo with fat mass and negative correlation between adiponectin with body mass and fat mass (Table 5).

Previously, it was shown that massive weight loss promoted an increase in adiponectin and adiponectin/leptin (A/L) ratio; additionally, a decrease in leptin levels and a reduction in exercise induced bronchospasm frequency and asthma-related symptoms, improving pro/anti-inflammatory adipokines. Furthermore, the leptin concentration was a predictor factor to explain changes in lung function, demonstrating the role of this adipokine in the inflammatory process, linking obesity and pulmonary disorders [7]. Therefore, another significant finding in the present investigation was a reduction in the prevalence of asthma from 16% to 0%. 

Interestingly, states of hyperleptinemia have also been implicated in the development of atherosclerosis [9]. Supporting this, at the beginning of the intervention, 75% of the analysed obese adolescents presented a hyperleptinemia state; after intervention 55% remained in this condition, but both prevalence and median values of this adipokine were significantly decreased as mentioned above. 

Although there was a significant improvement in the inflammatory state resulting from leptin reduction, leptin/adiponectin ratio, and increase in adiponectin, others important inflammatory markers such as TNF-*α*, resistin, and CRP were not reduced. A possible explanation could be the presence of a hyperleptinemia state since 55% of the adolescents remained with high levels of leptin, even after significant reduction in concentration, suggesting that the neuroendocrine system could interpose in inflammatory process. However the complete understanding of the interplay between neuroendocrine regulations of energy balance and inflammation needs to be explored further. Reinforcing this hypothesis, Corgosinho et al. showed a positive correlation between PAI-1 and some orexigenic neuropeptides, such as the NPY, MCH, and the NPY/AgRP ratios suggesting that the impairment in the control of energy balance acts in a dependent manner, involving inflammatory processes [35].

Some limitations need to be taken into account in interpretation of these findings. First, we had a dropout of 20% of the sample. The main reasons for dropping out in our study were financial and domestic, followed by education and job opportunities. Secondly, there is no control group in this study to compare with the normal states of cytokines and inflammation markers. Thirdly, a larger sample size is needed to better confirm the findings. However, the strengths of the current study include the assessment of a wider range of obesity comorbidities and parameters in a sample of obese adolescents undergoing interdisciplinary long-term therapy. 

Finally, it is important to observe that obesity is estimated to reduce average of life expectancy and is imposing a major economic burden on health insurance [16, 40, 41]. Increasing physical exercise and decreasing sedentary behaviour are a worldwide challenge. In fact, an American study aimed to increase physical activity among Latin adolescents through a school-based program has proved successful in decreasing sedentary behaviours. However the study group did not increase physical activity [42]. Together, the results call for more initiatives from both public and private health insurance to challenge not only the control of obesity *per se* but also the patterns of altered parameters which may impair the effect of multidisciplinary strategies in improving health. 

## 5. Conclusion

The multidisciplinary approach reduced not only obesity but also the prevalence of metabolic syndrome, NAFLD, asthma, inflammatory markers, and cardiovascular risk. Moreover, an improvement in the neuroendocrine regulation of energy balance was observed. Finally, the design of a type of therapy that favours better adherence is a challenge, considering the complexity of this disease and the possibility of increasing quality and life expectation. 

## Figures and Tables

**Table 1:** Prevalence of metabolic disorders associated with obesity.

Variables		Baseline (%)	After therapy (%)	*P* value
Metabolic syndrome factors (IDF classification)	Glucose	13	5	<0.001
	Insulin	23	4	<0.001
	Triglyceride	17	7	<0.001
	HDL-cholesterol	42	31	0.30
	Systolic blood pressure	26	5	<0.001
	Diastolic blood pressure	14	2	<0.001
	Waist circumference	62	14	<0.001

MS NAFLD	Metabolic syndrome	30	2	<0.001
	Nonalcoholic fatty liver disease	33	10	<0.001

Others complications	HOMA-IR	83	40	<0.001
	Hyperleptinemia	75	55	<0.001
	Asthma	16	0	<0.001
	Binge eating	6	2	<0.001

Statistical significance *P* ≤ 0.05; HOMA-IR: homeostasis model assessment insulin-index resistance; HLD: high-density lipoprotein; IDF: International Diabetes Federation; MS: metabolic syndrome; NAFLD: nonalcoholic fatty liver disease.

**Table 2:** Effects of interdisciplinary therapy in body composition.

Variables	Baseline	After therapy	*P* value	Δ value
Body mass (kg)	106.2 ± 16.2	94.4 ± 16.9	<0.001	−11.1 ± 7.7
Height (m)	1.68 ± 0.08	1.69 ± 0.08	0.07	0.009 ± 0.01
BMI (kg/m²)	37.0 ± 4.95	32.9 ± 5.32	<0.001	−4.27 ± 2.82
Fat mass (kg)	49.72 ± 11.66	36.5 ± 11.9	<0.001	−13.23 ± 7.02
Fat mass (%)	46.8 ± 5.70	38.0 ± 7.7	<0.001	−8.56 ± 4.58
Free fat mass (kg)	54.7 ± 10.4	57.0 ± 11.9	<0.001	2.24 ± 4.29
Free fat mass (%)	53.1 ± 5.7	61.9 ± 7.7	<0.001	8.74 ± 4.84
Visceral fat (cm)	4.5 ± 1.5	2.8 ± 1.3	<0.001	−1.60 ± 1.13
Subcutaneous fat (cm)	4.1 ± 0.8	3.2 ± 0.8	<0.001	−0.84 ± 0.80
Waist circumference (cm)	103.4 ± 10.7	94.9 ± 11.6	<0.001	−9.5 ± 18.32
Systolic blood pressure (mmHg)*	120 (100–190)	110 (100–140)	<0.001	−10.00 (−70–20)
Diastolic blood pressure (mmHg)*	80 (70–110)	70 (60–90)	<0.001	0.00 (−40–10)

*Nonparametric data; BMI: body mass index; statistical significance *P* ≤ 0.05.

**Table 3:** Effects of interdisciplinary therapy in metabolic profile (factors associated with metabolic syndrome definition by International Diabetes Federation (IDF)).

Variables	Baseline	After therapy	*P* value	Δ value
Waist circumference (cm)	103.4 ± 10.7	94.4 ± 11.6	<0.001	−9.5 ± 18.32
Glucose (mg/dL)	90.5 ± 7.8	89.7 ± 7.7	0.43	0.77 ± 8.60
Insulin (*µ*U/mL)	17.1 ± 8.0	12.0 ± 9.6	<0.001	−4.94 ± 10.50
HDL-cholesterol (mg/dL)	43.7 ± 8.6	45.3 ± 9.2	<0.001	1.80 ± 5.51
LDL-cholesterol (mg/dL)	102.4 ± 27.4	94.0 ± 24.0	<0.001	−9.98 ± 17.54
VLDL-cholesterol (mg/dL)	21.3 ± 9.4	17.8 ± 8.5	<0.001	−3.44 ± 8.06
Triglyceride (mg/dL)	111.2 ± 64.2	88.9 ± 42.6	<0.001	−21.27 ± 52.50
HOMA-IR	3.8 ± 2.0	2.7 ± 1.7	<0.001	−1.13 ± 3.00

HOMA-IR: homeostasis model assessment insulin-index resistance; HLD: high-density lipoprotein; LDL: low-density lipoprotein; VLDL: very-low-density lipoprotein. Reference values: glucose (60–110 mg/dL); insulin (<20 *μ*U/mL); HOMA-IR (<2.0); total cholesterol (<170 mg/dL); Triglyceride (33–129 mg/dL); HDL cholesterol (>38 mg/dL); LDL cholesterol (<130 mg/dL); VLDL cholesterol (10–50 mg/dL) (2); statistical significance *P* ≤ 0.05.

**Table 4:** Effects of interdisciplinary therapy in neuroendocrine regulation of energy balance and inflammatory profile.

Variables		Baseline	After therapy	*P* value	Δ value
Neuroendocrine regulation of energy balance	AgRP (ng/mL)	0.76 ± 1.45	0.55 ± 0.34	<0.001	0.05 ± 0.14
	NPY (ng/mL)	1.42 ± 1.75	1.55 ± 2.28	0.66	0.14 ± 2.82
	NPY/AgRP ratio*	0.41 (0.03–9.1)	0.42 (0.01–5.33)	0.07	0.02 (−2.47–2.86)
	MCH (ng/mL)	4.14 ± 2.42	4.95 ± 2.50	0.80	0.45 ± 1.81
	*α*-MSH (ng/mL)	0.99 ± 0.54	1.06 ± 0.75	0.03	0.10 ± 0.44
	Ghrelin (ng/mL)	1.10 ± 0.27	1.14 ± 0.24	0.40	0.02 ± 0.17
	Leptin (ng/mL)	42.59 ± 26.62	27.41 ± 20.48	<0.001	−19.77 ± 25.32

Inflammatory profile	Adiponectin (*µ*g/L)	6.61 ± 3.61	7.42 ± 4.61	<0.001	1.63 ± 2.69
	Lep/Adipo ratio*	5.7 (0.15–44.3)	3.7 (0.16–30.9)	<0.001	−10.48 (5.64–36.41)
	TNF-*α* (ng/mL)	44.31 ± 78.9	29.25 ± 41.45	0.23	−15.05 ± 52.03
	CRP (ng/mL)	6.38 ± 11.34	8.75 ± 14.87	0.48	1.82 ± 17.36
	Resistin (ng/mL)	12.45 ± 10.5	13.10 ± 10.3	0.71	0.22 ± 4.88
	PAI-1 (ng/mL)	13.4 ± 8.1	9.6 ± 8.3	<0.001	−3.94 ± 6.00
	interleukin-6 (IL-6)	58.3 ± 46.9	32.4 ± 21.7	0.05	−25.88 ± 49.85

*Nonparametric data; statistical significance *P* ≤ 0.05; AgRP: Agouti-related peptide; NPY: neuropeptide Y; MCH: melanin-concentrating hormones; *α*-MSH: *α*-melanocyte-stimulating hormone; Lep/Adipo ratio: leptin/adiponectin ratio; TNF-*α*: tumor necrosis factor-alpha; CRP: C-reactive protein; PAI-1: plasminogen activator inhibitor-1. Reference values: Leptin values between 1 and 20 ng/ml for males and between 4.9 and 24 ng/ml for females.

**Table 5:** Correlations analysis.

Variables (Δ values)		*R* value	*P* value
Fat mass (kg)	Free fat mass (%)	−0.75	0.001
	Adiponectin (*µ*g/L)	−0.72	0.001
	Lep/Adipo ratio	0.67	0.003
	Visceral fat (cm)	−0.57	0.871

Subcutaneous fat (cm)	Fat mass (%)	0.71	0.002
	Free fat mass (%)	−0.71	0.002

Adiponectin (*µ*g/L)	Body mass (kg)	−0.65	0.003
	Visceral fat (cm)	−0.20	0.571
	HOMA-IR	−0.20	0.873

PAI-1 (ng/mL)	Ghrelin (ng/mL)	0.68	0.002

Visceral fat (cm)	HOMA-IR	0.67	0.572
	Fat mass (%)	−0.28	0.421

Lep/Adipo ratio: leptin/adiponectin ratio; PAI-1: plasminogen activator inhibitor-1; HOMA-IR: homeostasis model assessment insulin index-resistance; statistical significance *P* ≤ 0.05.
